# Protection of Patinated Bronze with Long-Chain Phosphonic Acid/Organic Coating Combined System

**DOI:** 10.3390/ma16041660

**Published:** 2023-02-16

**Authors:** Dajana Mikić, Helena Otmačić Ćurković

**Affiliations:** Research Laboratory for Corrosion Engineering and Surface Protection, Faculty of Chemical Engineering and Technology, University of Zagreb, 10000 Zagreb, Croatia

**Keywords:** bronze patina, corrosion inhibitor, organic coating, cultural heritage protection, electrochemical measurements

## Abstract

Bronze cultural heritage in urban areas is susceptible to decay due to the significant amount of pollutants present in the air. This causes the dissolution of bronze and the patina on its surface. The efficient protection of outdoor bronze cultural heritage is still an unresolved problem. The aim of this work is to investigate 16-phosphonohexadecanoic acid as an environmentally friendly and non-toxic corrosion inhibitor for patinated bronze. The corrosion protection of sulphide-patinated bronze by phosphonic acid alone and in combination with acrylic coating Paraloid B-72 is examined. In order to achieve efficient corrosion protection, various parameters of the phosphonic acid application were studied. The efficiency of protection is examined by electrochemical impedance spectroscopy (EIS) during the immersion in simulated acid rain solution and after exposure to a corrosion chamber. It was found that the studied phosphonic acid provides corrosion protection to patinated bronze and significantly improves the protective properties of Paraloid B72. This was also confirmed by scanning electron microscopy (SEM) examination of the coating surface after exposure to a corrosive environment.

## 1. Introduction

The patina layer covering bronze cultural heritage has an important historical and aesthetic value but also provides corrosion protection to the underlying bronze substrate. In urban atmospheres, patinas directly exposed to rain are subjected to significant dissolution, which leads to the corrosion of underlying bronze [1,2,3,4,5,6,7,8]. Many studies have been conducted with the aim of understanding better the mechanism of bronze atmospheric corrosion and the influence of various factors such as alloy composition [4,5,6], presence of pollutants [2,9,10], and weathering conditions [7,11,12,13]. These factors influence not only the corrosion rate value but also the composition and structure of the patina layer. Even more, different weathering conditions (exposure to sun and rainfall) on the same object can lead to different corrosion mechanisms [8,14]. It was found that in urban atmospheres, patinas in unsheltered areas are unstable and are cyclically leached by rainwaters [8,15]. Chiavari et al. [16] found that in rainfall-runoff conditions (simulating unsheltered areas on the sculpture), the patina layer is thin, porous, and strongly enriched with insoluble Sn oxides, across which Cu, Zn, and Pb cations migrate; more than 90% of these cations are leached into the environment. Regular maintenance by cleaning and coating with a protective material is the most common approach used to preserve outdoor patinated bronze and prevent corrosion. However, the selection of applicable coatings is limited by the specific requirements for cultural heritage protection, requesting that the object’s visual appearance remains unchanged, simple application method, and coating reversibility [13,17,18]. The most commonly used protective materials are waxes, acrylic resins, and corrosion inhibitors, but all have some drawbacks. Waxes have a short lifetime, are not fully reversible, and are prone to dust accumulation [19,20]. The most common protective coatings for outdoor bronzes are acrylic coatings from the Paraloid family and Incralac (an acrylic resin solution containing corrosion inhibitor benzotriazole). They require regular maintenance, similar to waxes. Other drawbacks are the shiny appearance of the treated surface, the brittleness of the film, and difficult removal over time [20,21,22]. It has also been reported that Incralac is prone to biodeterioration [23,24]. In general, transparent coatings used for the protection of metallic cultural heritage can change color over time due to interactions with the environment and photooxidation mechanisms, which most often cause its yellowing [25]. Benzotriazole (BTA) is the most widely applied corrosion inhibitor for copper alloy heritage objects. It has been in use since the 1960s. The effectiveness of BTA has been proved for clean copper surfaces, but for corroded archaeological artifacts is questionable, especially for artifacts containing a chlorinated phase [18,26]. Today, BTA is also known to be toxic.

The metallic surfaces of cultural heritages differ significantly one from another in terms of the degree of corrosion, different types of corrosion products, and the presence of microorganisms. It can be challenging to achieve good coating adhesion on such surfaces, as not every protection is equally effective on every type of patina [27,28]. Numerous research studies are being conducted in order to develop more effective protection of bronze cultural heritages. The goal is to find protection that will be long-lasting, environmentally friendly, and non-toxic, respecting all the requirements of the restoration and conservation profession [18]. Great efforts are being made to find inhibitors that might replace BTA [29,30,31,32,33,34,35], improve the properties of existing coatings, or develop new coatings such as plasma polymers [36,37], fluoropolymers [37,38,39,40], silane-based polymers [17] or diamond-like carbon coating [41]. However, each of these coatings exhibits certain drawbacks. For example, plasma polymer coatings show reversible problems because they are not easy to remove [37]. A similar issue was reported for fluoropolymer coatings together with the low adhesion to metal [37]. The diamond-like carbon coating is deposited by plasma-enhanced chemical vapor, a procedure too demanding for practical application [37].

Despite the extensive scientific research on new types of coatings, those from the Paraloid family (Paraloid B-44 and B-72, Incralac) are still the most popular for archaeological or historical copper alloy objects [42], but they exhibit relatively short protection time. Thus several studies examined how to improve their protective properties and extend their lifetime.

Ntelia et al. [43] examined Paraloid B-72 in combination with SiO_2_ on brass to obtain superhydrophobic coating, but it had strong effects on the color of treated brass, which is not in accordance with the requirements of the restoration and conservation profession. Cano et al. [44] added commercial corrosion inhibitor additives to Paraloid B-72 to protect historic steel artifacts. This resulted in an initial increase in the protective properties of the coatings, but after artificial aging, the protective properties of the coatings were worse than those of Paraloid B-72 alone. In our previous studies, we investigated imidazole derivatives and BTA as corrosion inhibitors for different types of bronze patina and in combination with Paraloid B-44. The effectiveness of the protection depended significantly on the type of patina to which it was applied. It was found that inhibitors were only slightly effective on electrochemically patinated surfaces and had almost no effect on chemically patinated surfaces. Significant protection was achieved when inhibitors were added to the Paraloid B-44 solution, although protective effect decreased over time [45].

The aim of this work is to examine the possibility of using a long-chain phosphonic acid (16-phosphonohexadecanoic acid) as an environmentally friendly and non-toxic corrosion inhibitor for patinated bronze and to determine whether it can improve the protective properties of acrylic coating Paraloid B-72. It was proven that long-chain organic acids could protect clean metal surfaces [46,47], but until now, they were not investigated for the protection of pre-corroded/patinated surfaces and in combination with Paraloid B-72. Although corrosion inhibitors are often added into coating formulation, our preliminary studies showed that much better results are obtained when studied phosphonic acid is applied prior to Paraloid application. A similar approach has been explored in the conservation of ancient iron artifacts, where they were covered with silane and an external wax layer [48].

Studies are conducted on bronze covered with a sulphide patina layer since this type of patina is most commonly applied in practice as a foundry surface finish on bronze sculptures. Several methods of phosphonic acid surface application are evaluated: dip-coating, spraying, and brush application. In addition, the influence of application parameters such as drying temperature or the number of applications is evaluated.

The evaluation of the protective properties of studied coating systems was conducted on samples exposed either to continuous immersion in artificial rainwater or to dry-wet cycles in a corrosion chamber. For the moment, there are no standardized testing methods for the evaluation of outdoor bronze protective treatments, but among the most common are continuous immersion in corrosive solutions [3,40] and alternative exposure to wet-dry cycles, which are sometimes accompanied by UV radiation [40,49]. Continuous immersion leads to faster water penetration into the coating compared to cyclic exposure to wet conditions. Thus, the differences in protection between different coatings are sooner observed. On the other hand, exposure to wet-dry cycles better represents actual conditions during outdoor exposure. In previous studies [45], SO_2_ was introduced into the chamber as the most corrosive urban pollutant. However, SO_2_ concentrations have significantly decreased in the last decades, while NO_x_ concentrations in urban atmospheres are still high. For that reason, NO_2_ was introduced in a corrosion chamber in order to simulate the aggressively polluted urban atmosphere.

## 2. Materials and Methods

### 2.1. Materials

Experiments were performed on 0.5 cm thick discs, which were cut from a bronze rod with a 1.2 cm diameter, received from Strojopromet Ltd., Zagreb, Croatia. The composition of the bronze alloy is given in Table 1.

K_2_S_n_ (Sigma-Aldrich Corp.,Saint Louis, MO, USA) was used for bronze patination. 16-phosphonohexadecanoic acid (PA, 97%, Sigma-Aldrich Corp. Saint Louis, MO, USA) dissolved in ethanol (96% p.a., Lach-ner d.o.o., Zagreb, Croatia) was used for patinated bronze pretreatment. Paraloid B-72 (C.T.S., Altavilla Vicentina, Italia) dissolved in ethyl acetate (p.a., T.T.T., Sveta Nedjelja, Croatia) was used for bronze coating. Artificial rain was prepared by dissolving 0.2 g/L Na_2_NO_3_ (p.a., T.T.T., Sveta Nedjelja, Croatia), 0.2 g/L NaHCO_3_ (p.a., Kemika, Zagreb, Croatia), and 0.2 g/L Na_2_SO_4_ (p.a., Kemika, Zagreb, Croatia) in deionized water. The pH of the obtained artificial rain was adjusted to 5 with 0.5 M H_2_SO_4_. The conductivity of the artificial rain solution was 0.56 mS cm^−1^.

### 2.2. Sample Preparation

The bronze electrodes were abraded with 800, 1200, and 2500 grit emery paper, degreased with ethanol in an ultrasonic bath, and rinsed with deionized water. Then, they were placed in the furnace at 80 °C for half an hour to heat the entire surface of the bronze uniformly. K_2_S_n_ was dissolved in distilled water (1.25 g/50 mL) and heated to 80 °C. Samples were immersed in this solution for a few seconds immediately after removal from the furnace to obtain a brown sulphide patina. Afterward, the samples were polished with a sponge to remove the loosely attached patina. This procedure was repeated until the entire surface was covered with a patina. After preparation, samples were rinsed with deionized water and dried for three days at room temperature [50].

In order to determine the best way of PA application on the surface of patinated bronze, dip coating, spraying, and brushing methods were tested. In all cases, 1 mM PA ethanolic solution was used as this concentration was previously found to result in well protective film on bare bronze [46]. The PA film formation was conducted according to the experimental procedures presented in Table 2. The influence of the film drying temperature was examined, as well as the influence of adsorption temperature in the case of the dip coating method. Our previous studies have shown that these parameters have a significant impact on the protective properties of the phosphonic acid film [47,51].

Paraloid B-72 coating was applied either on bare patinated bronze samples or those covered by PA film. Dip coating and brushing were used as PA film application methods. For the brushing method, one and five applications of the PA solution onto the patina were tested, as well as film drying at room temperature and 80 °C. The dip coating method was conducted in a solution heated at 40 °C, followed by drying at 80 °C (Table 2). Afterward, Paraloid B-72 was applied with a brush from 15% *w/v* solution in ethyl acetate. After the coating application, samples were dried at room temperature for three days. The dry film thickness measured with PosiTector 6000 (DeFelsko) was 7 ± 2 μm. The thickness of the phosphonic acid film was determined by ellipsometry in our previous work [46], which is around 10 nm, and the thickness of the patina is around 1 μm.

### 2.3. Electrochemical Measurements

The electrochemical measurements were performed using a Bio-Logic SP-300 potentiostat in an acid rain solution. The measurements were conducted periodically during the three weeks of continuous immersion. Initial electrochemical measurements were performed after the sample had been immersed in the corrosive medium for 45 min to avoid the change of the open circuit potential (*E*_ocp_) during the acquisition of polarization or impedance data. The period of 45 min was sufficiently long for all studied samples to reach a steady state.

Electrochemical measurements were carried out in a three-electrode cell. The sample was set as the working electrode, and a saturated calomel electrode (SCE) and a Pt plate were used as the reference and counter electrodes. Potentiodynamic polarization (PDP) measurements were performed in a narrow (±20 mV vs. *E*_ocp_) potential range at the scan rate 0.166 mV s^−1^. Electrochemical impedance spectroscopy (EIS) measurements were conducted at *E*_ocp_ in the frequency range from 100 kHz–10 mHz with a 10 mV amplitude. At least three replicas of each type of sample are used for electrochemical measurements.

### 2.4. Surface Analysis

Surface analysis was performed with the scanning electron microscope (SEM) VEGA 3 TESCAN at an acceleration voltage of 10 kV and with an optical digital microscope Dino-Lite AM7515MT8A.

### 2.5. Exposure in a Corrosion Chamber

Accelerated aging was performed in a corrosion chamber with the introduction of NO_2_ for 14 days. Aging was conducted in the corrosion chamber CON 300-FLAIR CWC KES (VLM GmbH, Bielefeld, Germany) with alternating wet (8 h at 40 °C and 100% humidity) and dry (16 h at room temperature) cycles. One hour prior to wet cycles, NO_2_ was evolved in the chamber by a reaction of 1.1 mg of copper and 100 μL of 14 M HNO_3_ acid [50]. The maximum obtained level of NO_2_ was 1.4 ppm. The procedure for NO_2_ evolution was developed according to the study of Dorhout et al. [52]. After each dry period, the corrosion chamber was completely aerated.

## 3. Results and Discussion

### 3.1. Evaluation of Patinated Bronze Protection by PA

**Figure 1:**
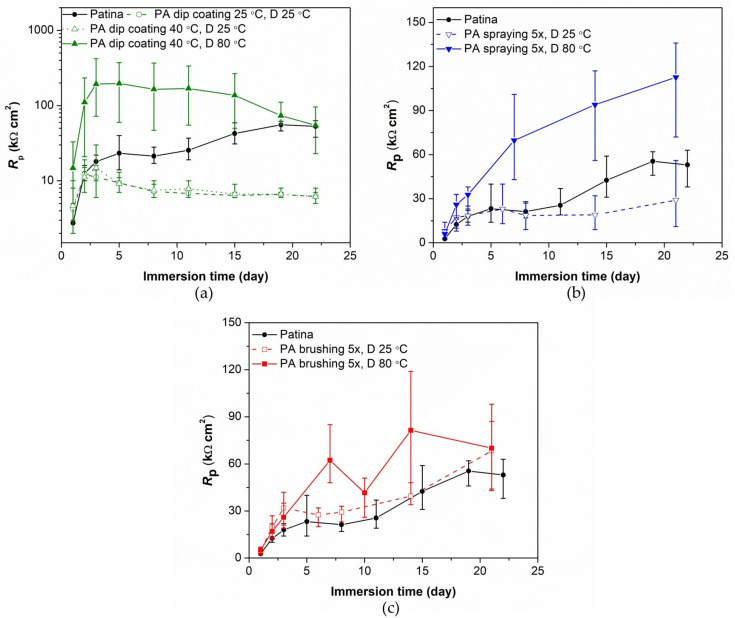
Polarization resistance as a function of immersion time in simulated acid rain for the unprotected patina and patina protected with phosphonic acid (PA) film obtained by (**a**) dip coating, (**b**) spraying, and (**c**) brushing method.

Firstly, the corrosion protection by PA alone (without Paraloid coating) was examined by electrochemical methods. Figure 1 shows a comparison of polarization resistance values for each PA application method and studied drying temperature in relation to unprotected patinated bronze samples. When examining the influence of drying temperature, it is clear that the films deposited by the dip coating and spraying method and dried at 80 °C provide better corrosion protection than those dried at room temperature. In the case of the brushing method, the influence of drying temperature on *R*_p_ values is less pronounced. It may be assumed that at an elevated temperature, molecules migrate more easily and become more evenly distributed over the surface, thus forming a more ordered film [53,54]. Elevated temperature also enhances the chemisorption of phosphonic acid on metal surfaces [55]. For this reason, it is not surprising that the films dried at 80 °C showed better protection and stability over time. On the contrary, the increase in dip coating adsorption temperature had no significant impact on the *R*_p_ value.

An increase in polarization resistance was observed for all samples after one day of immersion in a solution of simulated acid rain. For unprotected samples, an increase in *R*_p_ can be ascribed to the transformation of the patina layer from initially very reactive patina compounds to more stable patina, as observed in our previous work [50]. In the case of the protected samples, there is probably an additional influence of a reorganization of PA film into a more compact structure. This behavior was also observed for PA films formed on bare bronze [46]. On the other hand, for dip-coated and sprayed samples, dried at room temperature, after several days of immersion, *R*_p_ becomes lower than the blank sample. This can be ascribed to the PA desorption due to the weaker anchoring to the metal surface.

In general, the properties of the protective film depend on its porosity, thickness, and bonding strength between the PA anchoring group and the metal/patina surface. Different methods of film application used in this work may result in different amounts of adsorbed PA. In addition, it should be taken into account that patinated bronze represents a porous substrate and that the amount of the solution entering into the patina may vary between the methods. For example, the brushing method showed the smallest differences between the samples dried at room and elevated temperature. This could be the result of more uniform PA film application by brush, both on the outer surface and inner patina layer, than by the other two techniques. In such cases, the benefit of drying at elevated temperatures is mainly related to enhanced chemisorption and much less to film redistribution, such as in the case of the other two techniques. For this reason, it is not unusual that we obtained different levels of protection when different film application methods were used. In order to better understand the differences in the results between the examined application methods, electrochemical impedance spectroscopy measurements were conducted.

**Figure 2:**
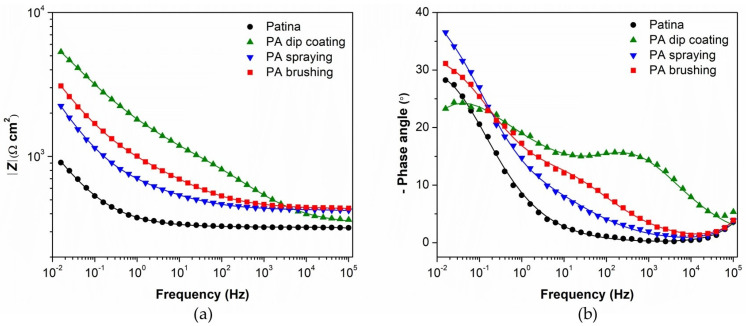
EIS—Bode plots of unprotected and PA-protected patinated bronze, measured on the first day of immersion: (**a**) impedance modulus, (**b**) phase angle. Symbols: experimental data; solid lines: modeled data according to electrical equivalent circuits given in Figure 4.

**Figure 3:**
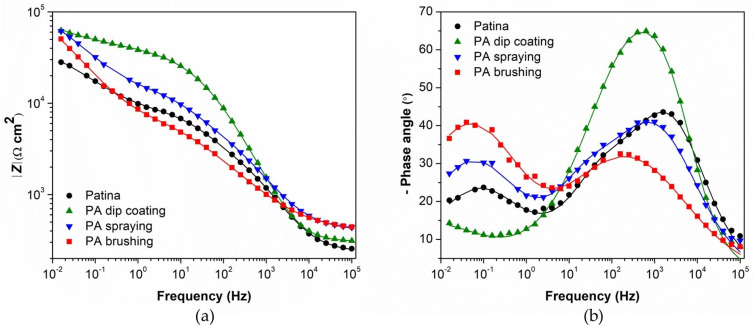
EIS—Bode plots of unprotected and PA-protected patinated bronze, measured on the 14th day of immersion: (**a**) impedance modulus, (**b**) phase angle. Symbols: experimental data; solid lines: modeled data according to electrical equivalent circuits given in Figure 4.

Electrochemical impedance spectroscopy measurements were conducted on samples with phosphonic acid films prepared by all three studied methods and dried at 80 °C since drying at lower temperatures did not result in satisfactory film properties. The Bode plot of the EIS results obtained on the first day of immersion is shown in Figure 2. From the impedance modulus plot (Figure 2a), it can be seen that the phosphonic acid films obtained by all three methods improved the corrosion resistance of the patinated bronze. After two weeks (Figure 3), the corrosion resistance of the bare patina and PA-treated patina increased, followed by a change in the phase angle plot. As the shape of the phase angle curve of bare and protected patinated bronze is changed in a quite similar way, it can be assumed that this is mainly the result of the transformations of the patina layer.

**Figure 4:**
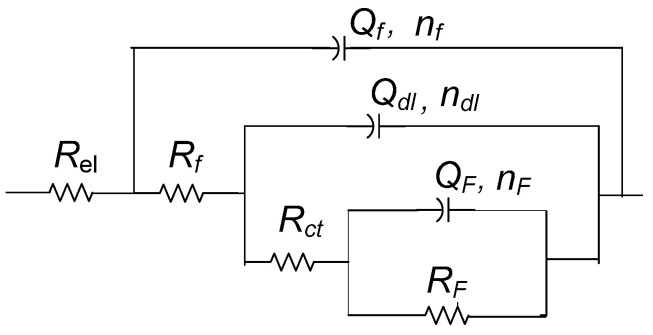
Equivalent electrical circuit used for fitting the impedance data.

In order to fit EIS spectra, it was necessary to use the model with three-time constants (Figure 4). Due to the inhomogeneity of studied surfaces, the capacitor element in electrical equivalent circuits was replaced with a constant phase element (CPE). The impedance of the CPE is defined as *Z*_CPE_ = 1/[*Q*(jω)*^n^*], where *Q* is the CPE’s frequency-independent parameter, which represents pure capacitance when *n* = 1. The proposed three-time constants EEC consist of *R*_f_ − *Q*_f_, *R*_ct_ − *Q*_dl,_ and *R*_F_ − *Q*_F_ couple. The high-frequency *R*_f_ − *Q*_f_ couple represents the capacitance and resistance of the non-reactive oxide film within the patina layer. *R*_ct_ − *Q*_dl_ couple represents the corrosion reaction at the metal substrate/solution interface, where *R*_ct_ is charge transfer resistance and *Q*_dl_ is the constant phase element representing the double layer capacitance. The low-frequency *R*_F_ − *Q*_F_ couple is allocated to the Faradaic resistance and Faradaic capacitance, implying the oxidation-reduction processes of the reactive patina layer [56]. The electrolyte resistance between the working and reference electrode is represented by *R*_el_.

Table 3 shows the results of the regression calculation. The EIS spectra for the first day (Figure 2b) reveal the existence of a poorly resolved capacitive behavior at the highest frequencies for all studied samples. For this reason, the values of *R*_f_ − *Q*_f_ couple cannot be reliably determined and are thus not shown in Table 3. For all samples, the *R*_ct_ values are quite low at the beginning of immersion in the acid rain solution, which could be due to the presence of corrosive S^2−^ ions inside the patina layer [50]. However, protected samples show higher *R*_ct_ values and lower *Q*_dl_ values compared to the unprotected sample. This confirms that phosphonic acid film slows down the corrosion process of bronze. The highest *R*_ct_ value is observed for the film obtained by dip coating. The probable explanation is that the longer contact of the sample with the PA solution, compared to the brushing and spraying method, resulted in the diffusion of a higher amount of PA molecules into the pores of the patina layer, reaching the bronze surface and protecting it more efficiently.

The *R*_F_ values of all samples also increased in time, while the *Q*_F_ values decreased, which can be attributed to the stabilization of the patina. Reactive species in the patina are transformed into less reactive compounds, which can now be observed as the *R*_f_ − *Q*_f_ couple in the high-frequency region. The resistances of the reactive (*R*_F_) and non-reactive patina layer (*R*_f_) are always higher in the presence of PA film. This confirms that PA effectively protects the bronze substrate, as well as the patina.

With the dip coating and spraying method, the darkening of the patina was observed by the naked eye after the application of the films. Given that in the case of the dip coating method, the patina is in longer contact with ethanol, in which PA is dissolved, it is possible that longer exposure to ethanol caused its darkening. On the other hand, it cannot be excluded that thicker PA surface films formed by dip coating and spraying resulted in color alteration. Thus, although the dip coating method initially resulted in the most protective PA film, from the practical point of view, it would be recommended to use the brush application method in order not to alter the visual appearance of the surface. In addition, *R*_p_ and EIS measurements confirmed that brushed samples exhibited less significant deterioration of protective properties during the aging in artificial acid rain compared to dip-coated samples.

### 3.2. Evaluation of Patinated Bronze Protection by PA/Paraloid System

**Figure 5:**
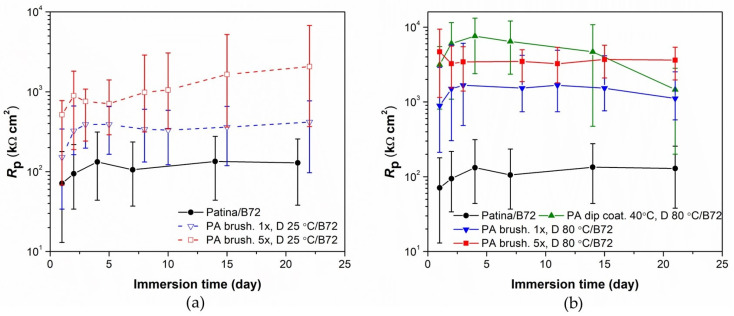
Polarization resistance as a function of immersion time in simulated acid rain for the patinated bronze samples coated with Paraloid B-72 alone or pretreated with (**a**) PA dried at room temperature and (**b**) PA dried at 80 °C.

The use of phosphonic acid as the only mean of corrosion protection of bronze does not provide long-term protection in an aggressive environment. Therefore, the combined protection system PA/Paraloid B-72 was examined. For this purpose, only brushing and dip coating were studied as methods for PA application, as described in Table 2. Such coated samples were also examined by polarization measurements in a narrow potential range during exposure to artificial acid rain solution. From the polarization resistance values (Figure 5), it is evident that the pretreatment of patinated bronze with PA has a beneficial effect on the protective properties of Paraloid B-72. The least pronounced improvement in *R*_p_ is obtained when the PA solution is applied only once, and the films are dried at room temperature. Given that the patina is porous, a single application of the acid solution is probably not sufficient to cover the entire surface. The increased number of film applications increased the amount of PA molecules on the outer surface and inside the patina layer, which resulted in higher corrosion protection. After three weeks of immersion in a solution of simulated acid rain, the samples, on which the PA solution was applied five times and the obtained films were dried at a temperature of 80 °C, exhibited the highest corrosion resistance, even higher than for the samples on which PA film was formed by the dip coating method. It is interesting to notice that for the dip-coated samples, longer exposure to a corrosive medium resulted in a decrease in polarization resistance, both in the case of the PA film alone or in combination with the Paraloid coating.

**Figure 6:**
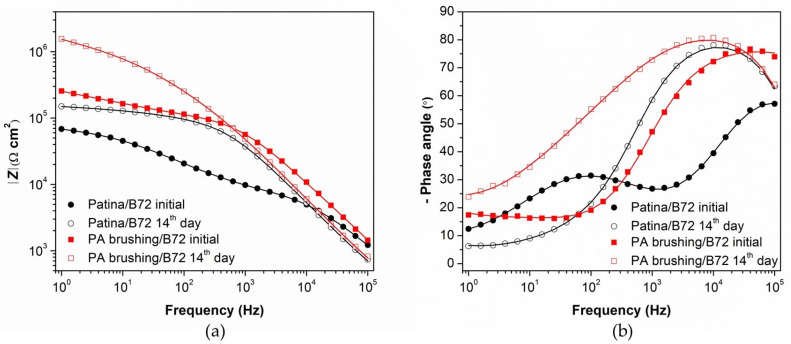
EIS—Bode plots for Paraloid B-72 and PA brushing 5×, D 80 °C /Paraloid B-72 protected patina measured first and 14th day of immersion: (**a**) impedance modulus, (**b**) phase angle. Symbols: experimental data; solid lines: modeled data according to electrical equivalent circuits given in Figure 7.

**Figure 7:**
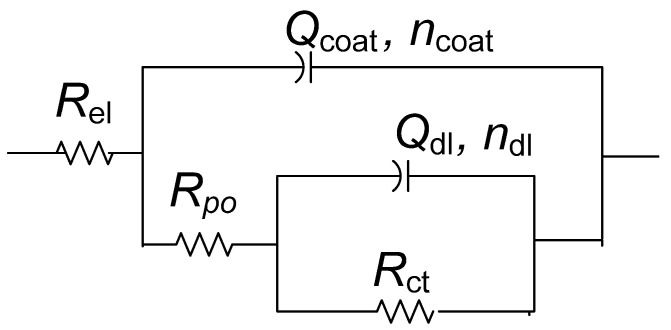
Equivalent electrical circuit used for fitting the impedance data in Figure 6.

In addition to polarization measurements, samples coated with Paraloid B–72 alone or combined with PA 5×, D 80 °C brushing pretreatment (the best result in Figure 5) were also characterized by electrochemical impedance spectroscopy (Figure 6). The EIS measurements were conducted in the frequency range from 100 kHz–10 mHz. The low-frequency region (1 Hz–10 mHz) exhibited a shape characteristic of diffusion impedance in pores, but it was difficult to determine the impedance parameters for this region unambiguously. Therefore, the EIS spectra in the frequency range from 100 kHz–1 Hz were examined by fitting to an electrical equivalent circuit model with two-time constants presented in Figure 7. Such a model is typically observed for coated surfaces, where *R*_po_ represents the resistance of the ionically conducting paths across the coating (“pores”) and *Q*_coat_ represents the capacitance of the coating [28]. *R*_ct_ − *Q*_dl_ couple represents the corrosion reaction at the metal substrate/solution interface, as described previously.

As can be seen from the Bode plot (Figure 6a), the impedance modulus values are higher after two weeks than on the first day of immersion, both for Paraloid B-72 and PA brushing 5×, D 80 °C /Paraloid B-72 protected patina. The *R*_po_ values (Table 4) are higher for patina treated with phosphonic acid. The phosphonic acid fills the pores of the patina and partially the pores of the coating, making it more difficult for the electrolyte to penetrate. The greatest influence of phosphonic acid on the improvement of the organic coating can be seen from *R*_ct_ values. They are much higher for patina treated with phosphonic acid. This confirms that the PA acid inhibits the dissolution of patinated bronze in the pores of the coating.

### 3.3. Surface Studies

**Figure 8:**
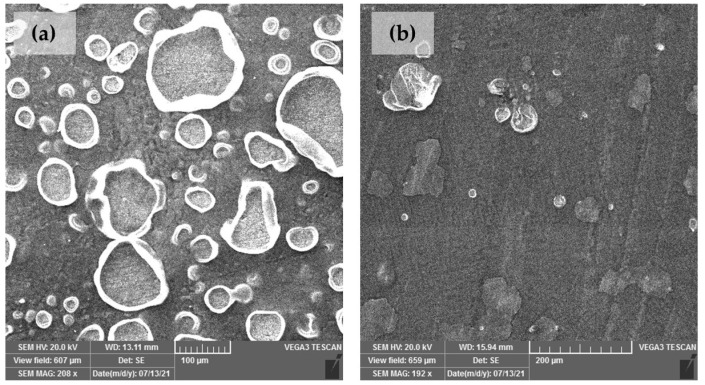
SEM images of patina coated with (**a**) Paraloid B-72 and (**b**) patina treated with PA brushing 5×, D 80 °C and coated with Paraloid B-72 after three weeks of immersion in acid rain solution.

Figure 8 shows SEM images of samples coated with Paraloid B-72 after being immersed in an acid rain solution for three weeks. The sample to which PA was applied (brushing 5×, D 80 °C) prior to coating has significantly fewer defects in the coating compared to the sample coated only with Paraloid B-72. From these images, it can be concluded that PA pretreatment can significantly improve the duration of Paraloid B-72 coating on patinated bronze. Any defect in the coating is a potential pathway for electrolyte penetration. Research on various substrates has shown that when electrolyte penetrates through the holes and reaches the metal/coating interface, rapid delamination of the coating occurs [57]. It is interesting to notice that EIS spectra of Paraloid B-72 coated samples (Figure 6) did not reveal the deterioration of protection by coating. This can be explained by the fact that the formation of defects in the coating is accompanied by a patina transformation in contact with the electrolyte. Thus the decrease in impedance due to the first process is compensated by the increase in impedance due to the second process occurring in the pores of the coating.

### 3.4. Exposure in a Corrosion Chamber

**Figure 9:**
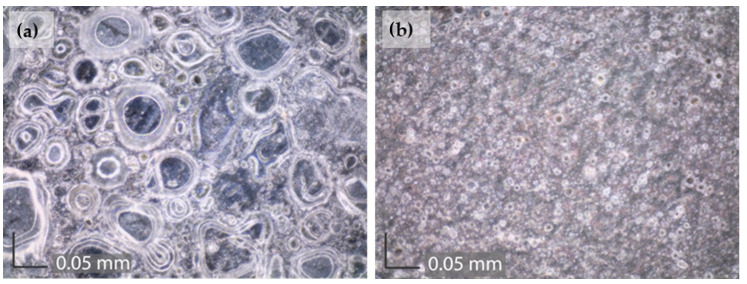
Optical microscopy images of (**a**) patina coated with Paraloid B-72 and (**b**) patina treated with PA and coated with Paraloid B-72 after two weeks in a corrosion test chamber.

The Paraloid B-72 coated samples, with or without the PA brushing (5×, D80 °C) pretreatment, were also exposed to the humidity corrosion test chamber with NO_2_. After two weeks of exposure, the samples were examined with an optical microscope at a magnification of 740× (Figure 9). The sample to which PA was applied showed significantly fewer defects in the coating. In the sample where the coating was applied directly to the patina, it is clearly visible that the entire coating is cracked. The obtained images are consistent with the SEM images obtained after exposure of the samples to acid rain solution and with results obtained with electrochemical measurements showing the beneficial effect of PA pretreatment on Paraloid B-72 protective properties.

## 4. Conclusions

In this work, a novel method of bronze cultural heritage protection, by surface treatment with selected long-chain phosphonic acid, was examined. The corrosion protection of sulphide patinated bronze with phosphonic acid films prepared by dip coating, spraying, and brushing was studied. The polarization resistance measurements in time showed that the elevated drying temperature of the films improved their protective properties and stability. PA films prepared by all three methods improved the corrosion resistance of patinated bronze if the drying step was conducted at 80 °C, while the PA films dried at room temperature exhibited protective properties only for the brushing method. The EIS results confirmed that such applied 16-phosphonohexadecanoic acid efficiently inhibited patina dissolution as well as the dissolution of the bronze substrate.

The corrosion protection of studied phosphonic acid was also tested in combination with acrylic coating Paraloid B-72. Electrochemical measurements showed a significant improvement in the protective properties of the coating when the patina was pretreated with phosphonic acid film dried at elevated temperatures. It was also found that a five-time brush application of PA resulted in significantly higher corrosion protection compared to a single application. In addition, the protective coating with PA applied by brush (5×, D 80 °C) exhibited a more stable protection level compared to the coating with PA applied by dip coating.

SEM images of the samples after three weeks of exposure to a simulated acid rain solution exhibited significantly fewer defects in the coating when the patina was pretreated with phosphonic acid film. Optical microscopy images of the samples after two weeks in the corrosion chamber also showed that the coating had significantly less deteriorated when the patina was pretreated with studied phosphonic acid.

The results of the studies conducted in this work clearly show that five-time brushing of PA solution accompanied by drying at 80 °C is an effective and simple pretreatment that significantly improves the efficiency and the durability of corrosion protection of sulphide patinated bronze by Paraloid B-72 coating.

These findings open the possibility of PA application in the field of bronze cultural heritage protection. However, additional issues, such as the dependence of protection level on the bronze composition and surface roughness, should be examined, which will be the focus of our further studies.

## Figures and Tables

**Table 1:** Composition of the bronze alloy.

Element	Cu	Sn	Pb	Ni	P	Zn	Rest
Wt. (%)	87.94	11.02	0.54	0.29	0.10	0.07	0.04

**Table 2:** Experimental conditions for PA film formation.

Method	Adsorption	Drying	Sample
Patina			
Dip coating	20 h at 25 °C	20 h at 25 °C	PA dip coating 25 °C, D 25 °C
	20 h at 40 °C	20 h at 25 °C	PA dip coating 40 °C, D 25 °C
	20 h at 40 °C	5 h at 80 °C	PA dip coating 40 °C, D 80 °C
Spraying	5× at room temp. *	20 h at 25 °C	PA spraying 5×, D 25 °C
	5× at room temp. *	5 h at 80 °C	PA spraying 5×, D 80 °C
Brushing	5× at room temp. *	20 h at 25 °C	PA brushing 5×, D 25 °C
	5× at room temp. *	5 h at 80 °C	PA brushing 5×, D 80 °C
Patina/Paraloid B-72			
Dip coating	20 h at 40 °C	5 h at 80 °C	PA dip coating 40 °C, D 80 °C/B72
Brushing	1× at room temp.	20 h at 25 °C	PA brushing 1×, D 25 °C/B72
	1× at room temp.	5 h at 80 °C	PA brushing 1×, D 80 °C/B72
	5× at room temp. *	20 h at 25 °C	PA brushing 5×, D 25 °C/B72
	5× at room temp. *	5 h at 80 °C	PA brushing 5×, D 80 °C/B72

* 30 min between each application.

**Table 3:** Impedance parameters obtained by fitting experimental data to selected equivalent circuits (Figure 4).

	*R*_f_ (kΩ cm^2^)	*Q*_f_ (µS S^n^ cm^−2^)	*n*_f_	*R*_ct_ (kΩ cm^2^)	*Q*_dl_ (µS S^n^ cm^−2^)	*n*_dl_	*R*_F_ (kΩ cm^2^)	*Q*_F_ (µS S^n^ cm^−2^)	*n*_F_
Patina									
1st day				0.14	4097	0.50	4.69	776	0.84
After 2 weeks	2.94	1.01	0.76	5.25	4.30	0.71	38	98	0.57
After 3 weeks	2.55	0.87	0.77	8.72	7.77	0.60	56	80	0.68
Patina/PA dip coating 40 °C, D 80 °C									
1st day				2.18	285	0.50	10	331	0.50
After 2 weeks	16	0.33	0.87	28	4.76	0.50	91	95	0.50
After 3 weeks	40	0.11	1	23	1.34	0.71	46	108	0.50
Patina/PA spraying 5×, D 80 °C									
1st day				0.38	696	0.50	33	1143	0.54
After 2 weeks	6.46	1.78	0.69	7.80	3.51	0.75	110	51	0.61
After 3 weeks	6.09	1.68	0.70	15	6.60	0.59	93	45	0.74
Patina/PA brushing 5×, 80 °C									
1st day				0.66	295	0.50	21	915	0.50
After 2 weeks	8.24	13.38	0.53	5.99	21	1	104	63	0.72
After 3 weeks	8.50	15.53	0.55	11.55	38	1	92	73	0.76

**Table 4:** Impedance parameters obtained by fitting experimental data to selected equivalent circuits (Figure 7).

	*R*_po_ (kΩ cm^2^)	*Q*_coat_ (nS S^n^ cm^−2^)	*n*_coat_	*R*_ct_ (kΩ cm^2^)	*Q*_dl_ (µS S^n^ cm^−2^)	*n*_dl_
Paraloid B-72						
1st day	8	20	0.82	66	1.19	0.60
After 2 weeks	77	6.64	0.93	73	0.86	0.50
After 3 weeks	76	8.58	0.93	89	0.60	0.55
PA brushing 5×, D 80 °C /Paraloid B-72						
1st day	97	6.00	0.87	215	1.15	0.50
After 2 weeks	128	5.04	0.94	1741	0.11	0.50
After 3 weeks	126	5.34	0.94	1844	0.13	0.50

## Data Availability

Data that support the findings of this study are available from the corresponding author upon reasonable request.
